# Paternal spatial training enhances offspring’s cognitive performance and synaptic plasticity in wild-type but not improve memory deficit in Alzheimer’s mice

**DOI:** 10.1038/s41598-017-01811-3

**Published:** 2017-05-08

**Authors:** Shujuan Zhang, Xiaoguang Li, Zhouyi Wang, Yanchao Liu, Yuan Gao, Lu Tan, Enjie Liu, Qiuzhi Zhou, Cheng Xu, Xin Wang, Gongping Liu, Haote Chen, Jian-Zhi Wang

**Affiliations:** 1 Pathophysiology Department, School of Basic Medicine and the Collaborative Innovation Center for Brain Science, Key Laboratory of Ministry of Education of China for Neurological Disorders, Tongji Medical College, Huazhong University of Science and Technology, Wuhan, 430030 China; 2 Neurology Department, Center Hospital of Huang-gang City, Huang-gang, Hubei Province China; 3 Key Laboratory for Molecular Diagnosis of Hubei Province, The Central Hospital of Wuhan, Tongji Medical College, Huazhong University of Science and Technology, Wuhan, 430014 China; 4 Co-innovation Center of Neuroregeneration, Nantong University, Nantong, 226001 China

## Abstract

Recent studies suggest that spatial training can maintain associative memory capacity in Tg2576 mice, but it is not known whether the beneficial effects can be inherited from the trained fathers to their offspring. Here, we exposed male wild-type and male 3XTg Alzheimer disease (AD) mice (3-m old) respectively to spatial training for one week and assessed the transgenerational effects in the F1 offspring when they were grown to 7-m old. We found that the paternal spatial training significantly enhanced progeny’s spatial cognitive performance and synaptic transmission in wild-type mice. Among several synapse- or memory-associated proteins, we observed that the expression level of synaptotagmin 1 (SYT1) was significantly increased in the hippocampus of the paternally trained-offspring. Paternal training increased histone acetylation at the promoter of SYT1 in both fathers’ and the offspring’s hippocampus, and as well as in the fathers’ sperm. Finally, paternal spatial training for one week did not improve memory and synaptic plasticity in 3XTg AD F1 offspring. Our findings suggest paternal spatial training for one week benefits the offspring’s cognitive performance in wild-type mice with the mechanisms involving an enhanced transgenerational histone acetylation at SYT1 promoter.

## Introduction

Physical training^1–4^ and mental^5–8^ or physiological stimulations^9^ to parents not only influence the individuals *per se* ^1, 2, 4, 10^, but also affect the behavioral performance, emotion, and cognitive functions of their children^5–7, 9^. For example, paternal odor fear conditioning enhances the offspring’s behavioral sensitivity to the F0-conditioned smell with an increasing neuroanatomical manifestation of the Olfr151 pathway^9^. In human, maternal tristimania during pregnancy augments risk of psychiatric disorders in the offspring, especially in female fetuses^11^. In an animal study, fathers’ MSUS (maternal separation combined with maternal stress) improves goal-directed behaviors and behavioral flexibility in the adult offspring, and this effect is demonstrated by epigenetic transformations including histone posttranslational modifications at the mineralocorticoid receptor (MR) gene promoter and decreased MR expression in the hippocampus^12^. However, the sole effects of fathers’ experience on offspring have seldom been studied.

Alzheimer’s disease (AD) is the most common neurodegenerative disorder characterized pathologically by amyloidogenesis and neurofibrillary degeneration^13, 14^ and clinically memory deterioration started by spatial memory loss^15, 16^. To date, there is no efficient cure for this devastating disorder. Recent studies suggest that spatial training can maintain associative memory capacity with enhancement of dendrite ramification and spine formation in Tg2576 mice, a recognized model of AD^17, 18^, but it is not known whether the beneficial effects can be inherited from the trained parents to their offspring.

In the present study, we investigated whether paternal spatial training could improve the cognitive function of the progeny in wild-type mice and in 3XTg mice, a well-recognized transgenic model of AD that contains both Aβ and tau pathologies with memory deficits at early age^19^.

## Results

### Father’s spatial training improves spatial memory with enhanced hippocampal synaptic transmission in 129s F1 offspring

**Figure 1:**
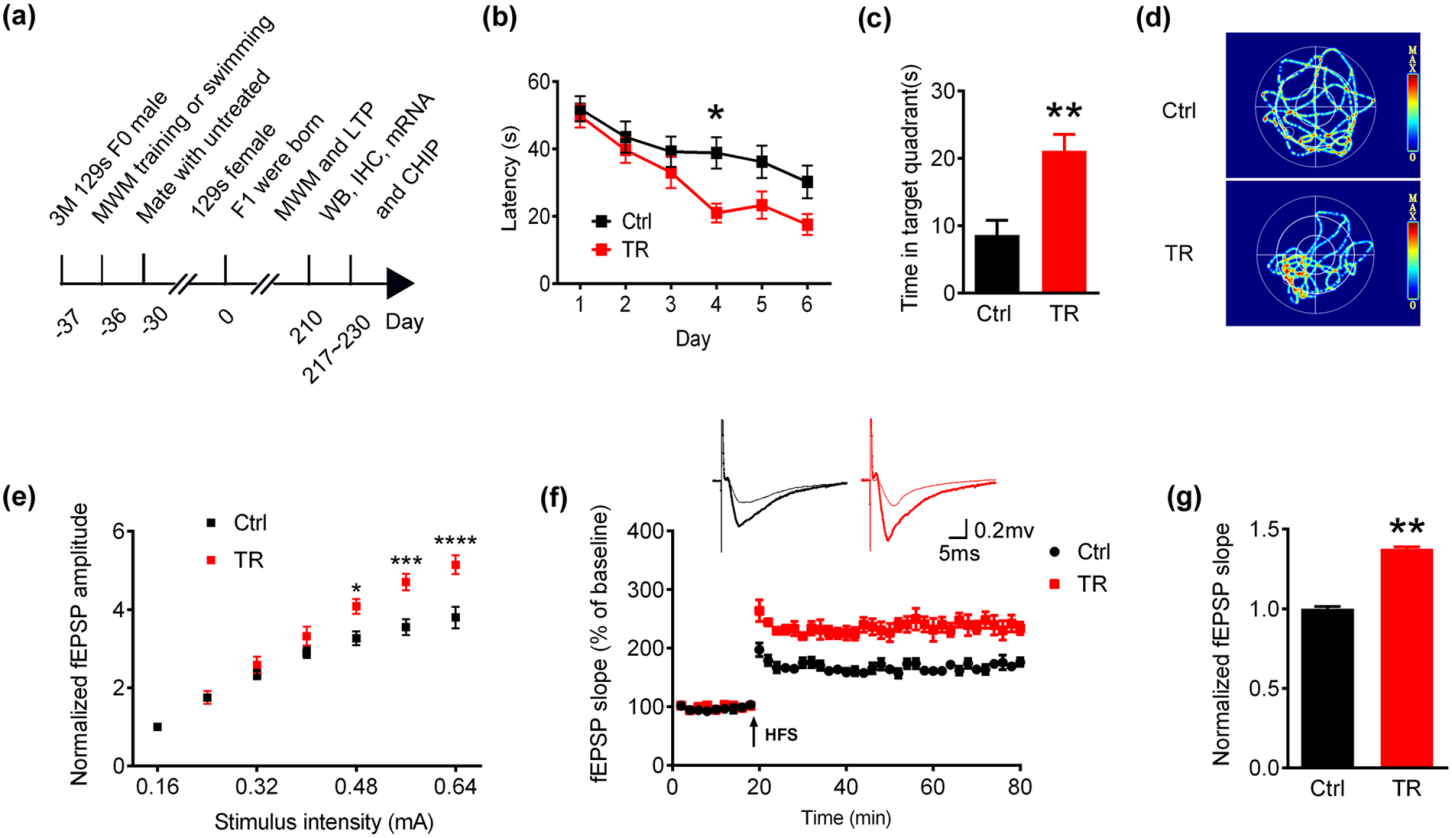
Paternal spatial training improves cognitive performance and synaptic transmission in 129s F1 offspring. (**a**) Spatial training paradigm and the experimental procedures. The birth of F1 offspring was defined as day zero. 37 days ago, F0 males were collected and engaged to Morris water maze (MWM) training or swimming (control). Those who behaved well were selected to mate with untreated females after MWM. The F1 offspring from different cages were bred under the same maintenance. When offspring grew up to 7-m old, they were conducted with MWM, Electrophysiological analysis (LTP), Western blotting (WB), Immunohistochemistry (IHC), mRNA extraction, and Chromatin immunoprecipitation (ChIP). (**b**) Escape latency to find the hidden platform in Morris water maze during 6 days training (two-way ANOVA row factor, F_5,229_ = 10.72, p < 0.0001; Bonferroni post hoc tests, *p < 0.05, n = 8 per group). (**c**) Quantitative analysis of time spent in the target quadrants measured at day 8 after removed the platform. Bar graphs show mean ± SEM (**p < 0.01, two-tail t-test, n = 6~8 per group). (**d**) The swimming pathway in the maze recorded at day 8 after removed the platform. (**e**) The Input/Output curve of the fEPSP recorded at cornu ammonis 1 (CA1) pyramidal neurons, normalized by fEPSP amplitude induced by minimum stimulation intensity (two-way ANOVA row factor, F_6,70_ = 137.4, p < 0.0001; Bonferroni post hoc tests, *p < 0.05, *** p < 0.001, ****p < 0.0001, 5 slices from 3 mice per group). (**f**) The slope of fEPSP after HFS, normalized by the fEPSP slope before HFS (the baseline). Arrow indicates the onset of HFS (100 Hz, 1 s duration). (**g**) Quantitative analysis of fEPSP slope. 6 values (fEPSP slopes against the baseline) around 60 min in each group were took for quantitative analysis. Bar graphs show mean ± SEM (**p < 0.01, two-tail t-test, 4~5 slices from 3 mice per group).

We trained 3-m old 129s male mice (F0) by Morris water maze (MWM) for 1 week (Supplementary Fig. 1a) and swimming with no training males were used as control, these mice were then mated with untreated 129s females (F0), finally the spatial learning and memory of their offspring (F1) at 7-m old was measured by MWM (Fig. 1a). Spatial training improved hippocampus-dependent fear memory shown by the increased freezing time in trained 129s fathers (Supplementary Fig. 1b). Moreover, compared with control F1 offspring (Ctrl), the paternally trained F1 offspring (TR) displayed significantly shorter escape latency during 6 days learning trails (Fig. 1b) and longer duration in the target quadrants during memory test by removing the platform at day 8 (Fig. 1c and d). These data suggest that spatial training in fathers can improve their offspring’s spatial learning and memory.

To verify the mechanisms underlying the ameliorated memory on offspring (F1) mice, we measured synaptic transmission on CA1 pyramidal neurons by electrophysiological recording on *ex vivo* acute brain slices. The trained group showed greater field excitatory postsynaptic potentials (fEPSP) amplitude demonstrated in I/O curves than Ctrl group (Fig. 1e). Moreover, 3 traces of high frequency stimulation (HFS) followed by baseline evoking significantly augmented long term potentiation (LTP) in the trained group compared with Ctrl group (Fig. 1f and g). These data indicate that spatial training to the wild-type fathers (F0) can improve synaptic plasticity and learning memory function in their F1 offspring.

### Father’s spatial training increases presynaptic synaptotagmin in 129s F1 offspring

**Figure 2:**
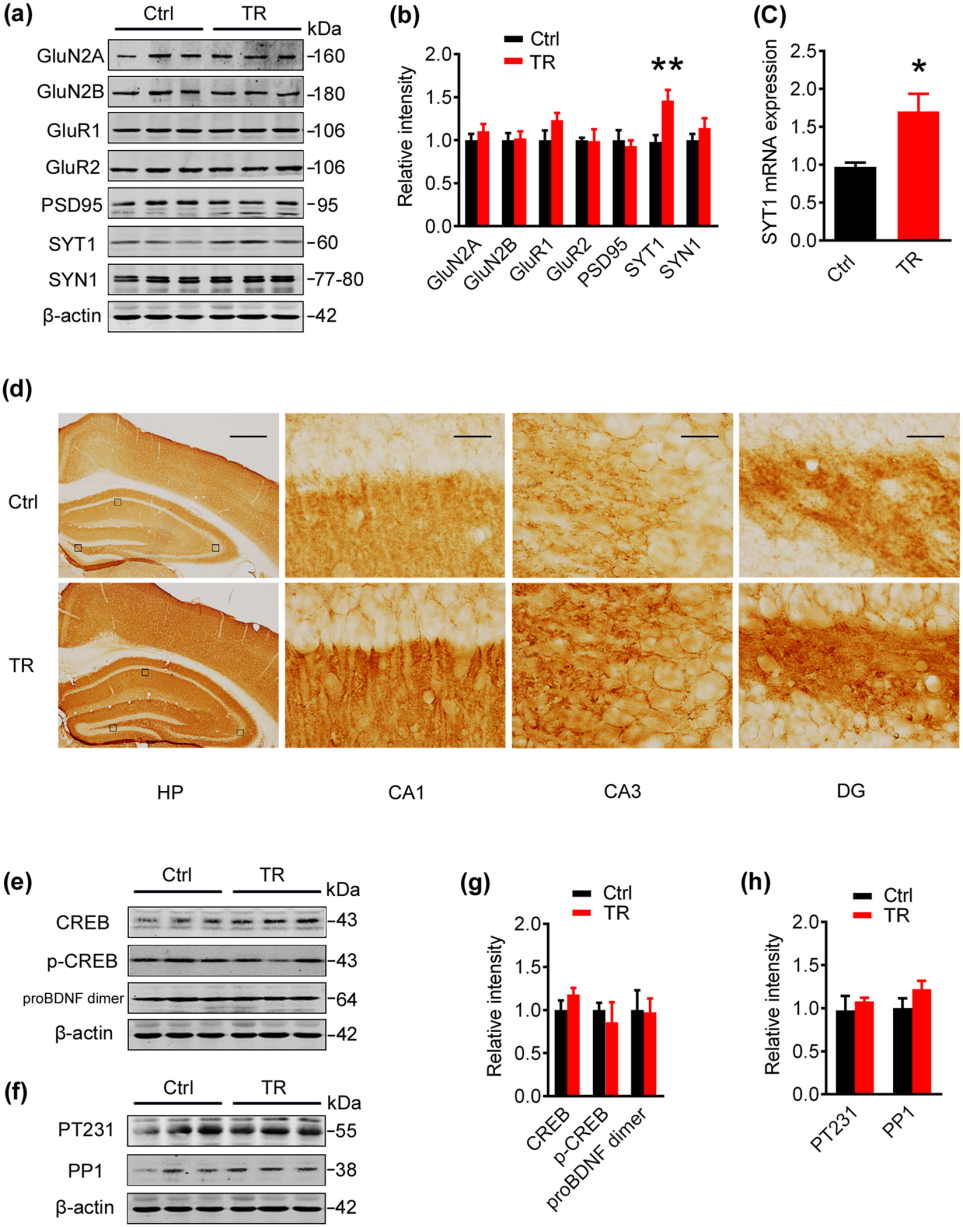
Father’s spatial training increases synaptotagmin 1 in 129s F1 offspring. (**a,b**) Father’s spatial training increased protein level of SYT1 in 7-m 129s F1 offspring measured by Western blotting. (**c**) Father’s spatial training increased mRNA level of SYT1 in 7-m 129s F1 offspring measured by quantitative RT–PCR. Bar graphs show mean ± SEM (*p < 0.05, two-tail t-test, n = 5~6 per group). (**d**) Immunohistochemical staining of SYT1 in cortex and hippocampus of 7-m 129s F1 brain slices. The HP (hippocampus) panel: scale bar = 50 μm; the enlarged CA1 (cornu ammonis 1), CA3 (cornu ammonis 3) and DG (dentate gyrus): scale bar  = 2 μm. (**e,g**) Expression level of CREB, p-CREB and proBDNF dimer in 7-m 129s F1 offspring measured by Western blotting. (**f,h**) Levels of phosphorylated tau (PT231) and protein phosphatase-1 (PP1) in 7-m 129s F1 offspring measured by Western blotting. (**a,b,e–h**) The blot images were cropped to display and quantitifed by the Odyssey Infrared Imaging System (Licor biosciences, Lincoln, NE, USA). Bar graphs show mean ± SEM. (**p < 0.01, two-tail t-test, pre-synaptic SYT1: n = 6 per group; the rest of synapse- or memory-associated proteins: n = 3 per group).

To explore the molecular mechanisms, we measured the levels of synaptic proteins in 7-m old F1 offspring by Western blotting. Among various synapse-associated proteins, including N-methyl-aspartate receptor 2A and 2B subunits (GluN2A, GluN2B), glutamate AMPA receptor A1 and A2 subunits (GluR1, GluR2), postsynaptic density protein-95 (PSD95), synapsin 1 (SYN1) and synaptotagmin1 (SYT1), we only found a significant increase of SYT1 (Fig. 2a and b), which is a presynaptic protein involved in vesicle docking and the Ca^2+^-evoked synaptic vesicle fusion^20, 21^. The increases of SYT1 mRNA and protein in 7-m old mice were also detected by RT-PCR, immunohistochemical/immunofluorescence staining (Fig. 2c,d and Supplementary Fig. 2a).

Previous studies showed that cAMP response element binding protein-brain derived neurotrophic factor (CREB-BDNF) pathway was modulated across generations^5, 22–24^, while we did not see significant change of CREB-BDNF in the F1 offspring (Fig. 2e and g). As PP1 acts as memory suppressor *via* stimulating CREB dephosphorylation and attenuating CREB-dependent gene expression^25^, we tested protein phosphatase-1 (PP1) level and its substrate phosphorylated tau (PT231), and no significant changes were found (Fig. 2f and h). These data together suggest that father’s spatial training can only selectively increase SYT1 among several synapse- or memory-associated proteins in the F1 offspring.

### Father’s spatial training augments histone acetylation on SYT1 promoter in hippocampus of the 129s F1 offspring

**Figure 3:**
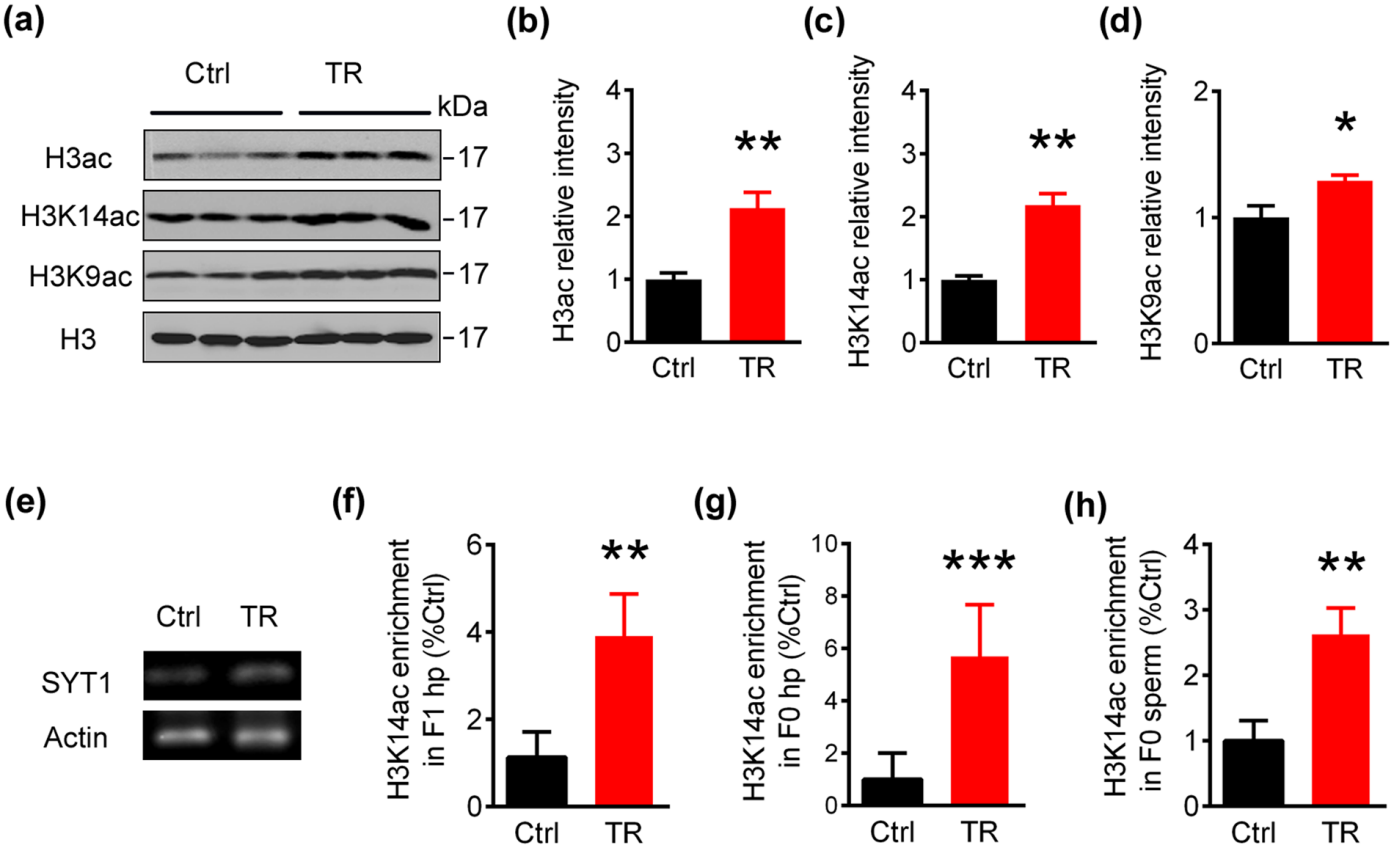
Paternal spatial training induces transgenerational augmentation of histone acetylation at SYT1 promoter regions in 129s F1 offspring. (**a–d**) Acetylated H3 (H3ac), acetylated H3K14 (H3K14ac) and acetylated H3K9 (H3K9ac) level in hippocampus of 7-m 129s F1 offspring normalized by histone 3 (H3) measured by Western blotting. The blot images were cropped to display and quantitifed by the Odyssey Infrared Imaging System (Licor biosciences, Lincoln, NE, USA). Bar graphs show mean ± SEM (*p < 0.05, **p < 0.01, two-tail t-test, H3ac: n = 6 per group; H3K14ac and H3K9ac: n = 3 per group). (**e,f**) Level of the acetylated H3K14 associated with SYT1 promoter in 7-m old F1 offspring’s hippocampal extracts measured by chromatin immunoprecipitation assay (ChIP). β-actin was probed as loading control. Bar graphs show mean ± SEM (**p < 0.01, two-tail t-test, n = 6 per group). (**g**) Level of the acetylated H3K14 associated with SYT1 promoter in 3-m fathers’ hippocampal extracts measured by ChIP, normalized against β-actin. Bar graphs show mean ± SEM (***p < 0.001, two-tail t-test, Ctrl group: n = 8, TR group: n = 6). (**h**) Level of the acetylated H3K14 associated with SYT1 promoter in 3-m fathers’ sperm extracts measured by ChIP, normalized against β-actin. Bar graphs show mean ± SEM (**p < 0.01, two-tail t-test, n = 6 per group).

Previous studies suggest that histone acetylation exerts long-lasting effects on modulating genes expression through lives^26–29^, even across generations^8, 30–32^. Therefore, we detected histone acetylation level in the offspring’s hippocampus by Western blotting. A remarkably increased total acetylated H3 (H3ac), the acetylated H3K14 (H3K14ac) and the acetylated H3K9 (H3K9ac) were shown in the trained offspring compared with the Ctrl (Fig. 3a–d).

To further validate the relationship between the upregulated SYT1 expression and the increased histone acetylation in trained group, we did chromatin immunoprecipitation assay (ChIP) in hippocampal extracts of the 7-m old F1 offspring. Compared with Ctrl group, the trained group showed increased association between SYT1 promoter gene and the acetylated H3K14 (Fig. 3e and f). These data suggest that the increased H3K14 acetylation at SYT1 promoter may underlie the upregulated mRNA and protein expression of SYT1 in F1 after father’s training.

### Spatial training enhances histone acetylation at SYT1 promoter in 129s fathers’ hippocampus and sperms

To trace how the fathers’ spatial training can translate into the offspring’s SYT1 upregulation, we conducted the same ChIP assay of SYT1 and acetylated H3K14 in hippocampus and sperms of the trained fathers (F0). Interestingly, a significant increase of H3K14 acetylation at SYT1 promoter was also detected in both hippocampus and sperms of the trained fathers compared with the Ctrl (Fig. 3g and h). These data imply the transgenerational influence of the trained fathers to their offspring.

### Father’s spatial training for one week does not improve memory and synaptic plasticity in 3XTg F1 offspring

**Figure 4:**
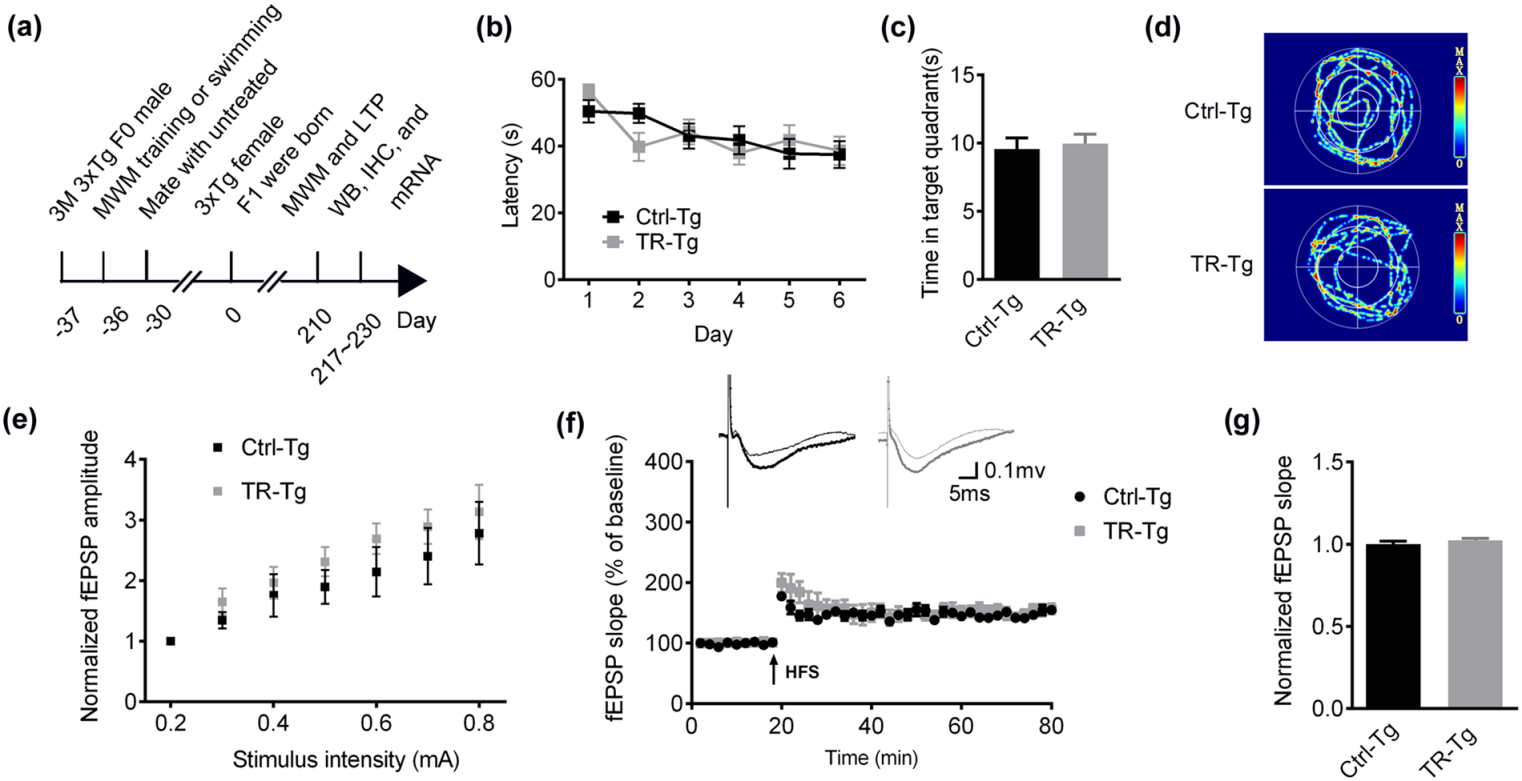
Paternal spatial training for 1 week does not improve cognitive performance and synaptic transmission in 3XTg F1 offspring. (**a**) Spatial training paradigm and the experimental procedures. The birth of F1 offspring was defined as day zero. 37 days ago, F0 males were collected and engaged to Morris water maze (MWM) training or swimming (control). Those who behaved well were selected to mate with untreated females after MWM. The F1 offspring from different cages were bred under the same maintenance. When offspring grew up to 7-m old, they were conducted with MWM, Electrophysiological analysis (LTP), Western blotting (WB), Immunohistochemistry (IHC) and mRNA extraction. (**b**) Escape latency to find the hidden platform in MWM during 6 days training trials (two-way ANOVA row factor, F_5,323_ = 4.395, p = 0.0007; Bonferroni post hoc tests, n.s, n = 9 ~10 per group). (**c**) Quantitative analysis of time spent in the target quadrants. Bar graphs show mean ± SEM (two-tail t-test, n.s, n = 7~10 per group). (**d**) The swimming pathway in the maze recorded at day 8 after removed the platform. (**e**) The Input/Output (I/O) curve of the fEPSP in CA1 pyramidal neurons, normalized by fEPSP amplitude induced by minimum stimulation intensity (two-way ANOVA row factor, F_6,42_ = 9.280, p < 0.0001; Bonferroni post hoc tests, n.s, 4 slices from 3 mice per group). (**f**) The slope of fEPSP after HFS normalized against the fEPSP slope before HFS (the baseline). Arrow indicates the onset of HFS (100 Hz, 1s duration). (**g**) Quantitative analysis of fEPSP slope. 6 values (fEPSP slopes against the baseline) around 60 min in each group were took for quantitative analysis. Bar graphs show mean ± SEM (two-tail t-test, n.s, 5 slices from 3 mice per group).

**Figure 5:**
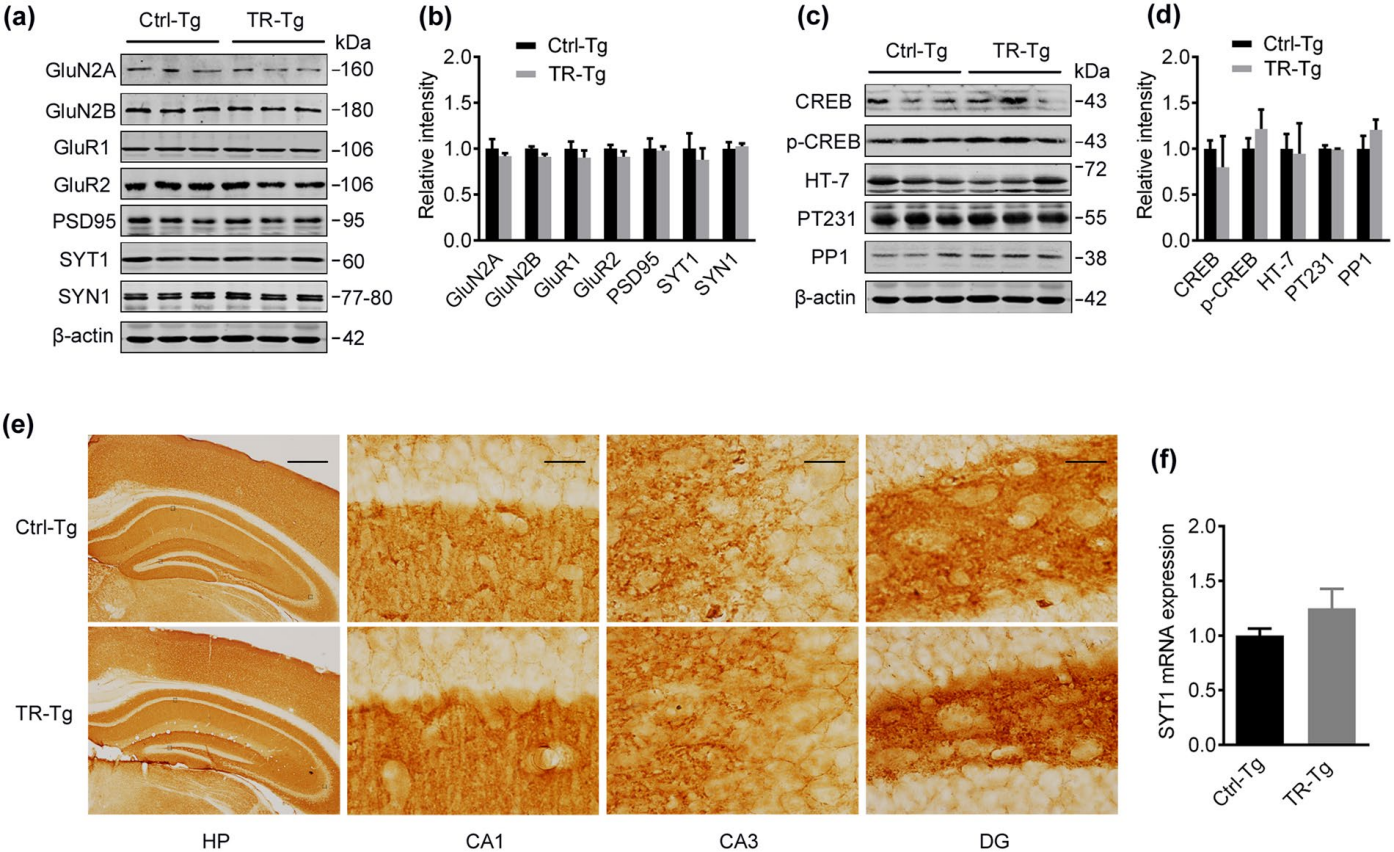
Paternal spatial training for 1 week does not improve synapse- or memory- associated proteins in 3XTg F1 offspring. (**a,b**) Synapse-associated proteins in 7-m 3XTg F1 offspring measured by Western blotting. (**c,d**) Memory-associated proteins (CREB, p-CREB), human tau (HT-7), phosphorylated tau (PT231) and PP1 in hippocampus of 7-m 3XTg offspring measured by Western blotting. The blot images in (**a–d**) were cropped to display and quantitifed by the Odyssey Infrared Imaging System (Licor biosciences, Lincoln, NE, USA). Bar graphs show mean ± SEM. (two-tail t-test, n.s, n = 3 per group). (**e**) Immunohistochemical staining of SYT1 in cortex and hippocampus of 7-m 3XTg offspring brain slices. The HP panel: scale bar = 50 μm; the enlarged CA1, CA3 and DG: scale bar  = 2 μm. (**f**) SYT1 mRNA level in hippocampus of 7-m 3XTg offspring measured by quantitative RT-PCR. Bar graphs show mean ± SEM (two-tail t-test, n.s, n = 3 per group).

By using the same training and mating paradigms, we trained 3-m old male 3XTg mice for 6 days in MWM (Supplementary Fig. 1a) and swimming males were used as control, and then mated them with 3XTg untrained females, and their F1 offspring at 7-m old were tested for spatial memory and synaptic plasticity (Fig. 4a). Although the improvement of fear memory was shown in trained 3XTg fathers (Supplementary Fig. 1c), we did not observe any significant improvement of spatial learning and memory in the trained 3XTg F1 offspring compared with the Ctrl-Tg group (Fig. 4b–d). Compared with the Ctrl-3XTg group, spatial training did not significantly change LTP (Fig. 4e–g) or the synaptic-associated proteins (Fig. 5a–d) in F1 offspring. The immunohistochemical/immunofluorescence staining and qPCR assay did not show changes of SYT1 protein and mRNA in the 7-m trained 3XTg F1 offspring (Fig. 5e,f and Supplementary Fig. 2b). These data suggest that paternal spatial training for 1 week may be not enough to preserve the cognitive function in AD mice.

### Father’s spatial training for one week does not promote histone acetylation in hippocampus of the 3XTg F1 offspring

**Figure 6:**
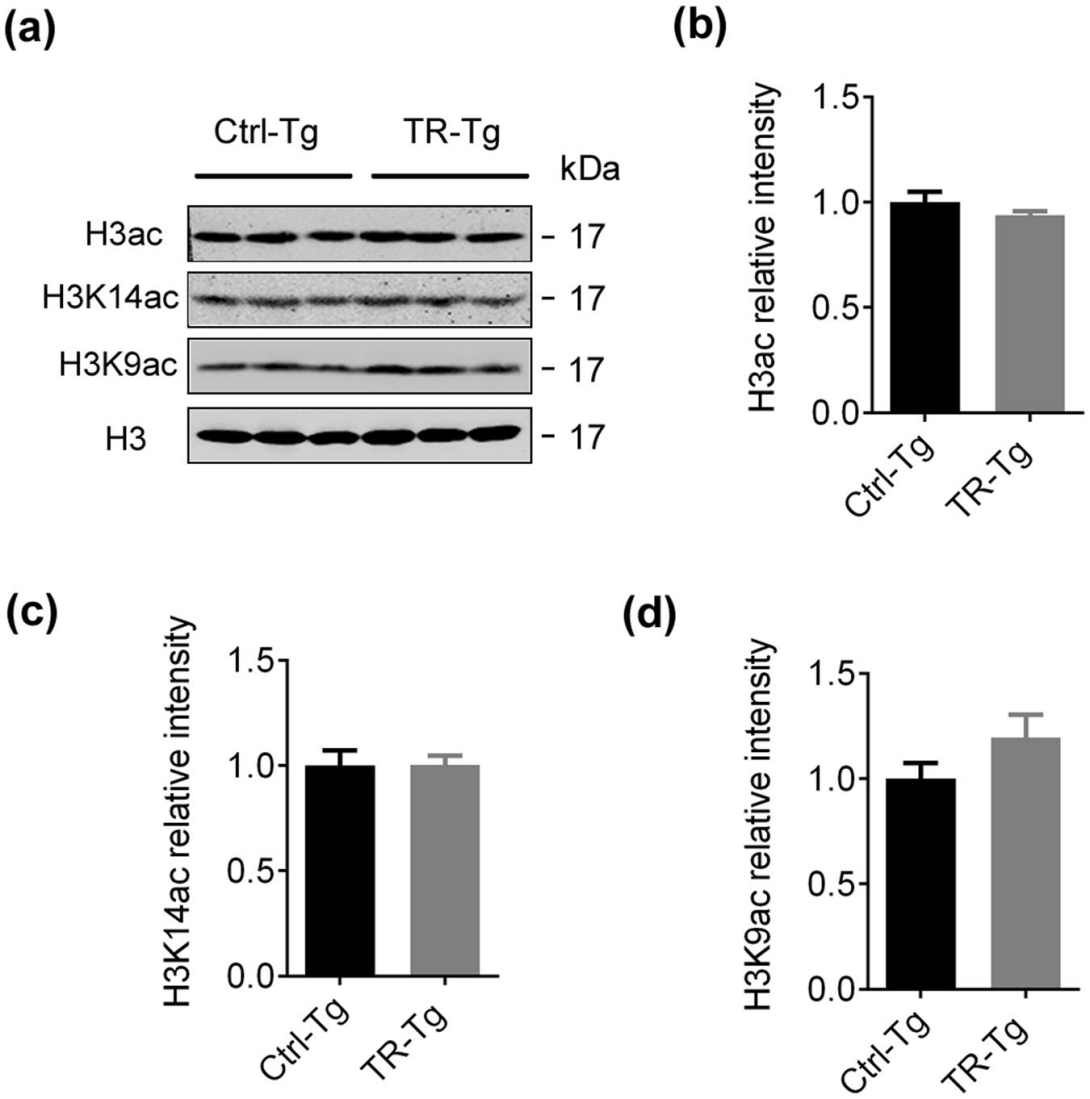
Paternal spatial training for 1 week does not induce transgenerational augmentation of histone acetylation in hippocampus in 3XTg offspring. (**a–d**) Acetylated H3 (H3ac), acetylated H3K14 (H3K14ac) and acetylated H3K9 (H3K9ac) level in hippocampus of 7-m 3XTg F1 offspring normalized by histone 3 (H3) measured by Western blotting. The blot images were cropped to display and quantitifed by the Odyssey Infrared Imaging System (Licor biosciences, Lincoln, NE, USA). Bar graphs show mean ± SEM. (two-tail t-test, n.s, n = 3 per group).

We also measured histone acetylation level in the 3XTg F1 offspring’s hippocampus by Western blotting. No significant differences were detected in total acetylated H3 (H3ac), the acetylated H3K14 (H3K14ac) and the acetylated H3K9 (H3K9ac) in the trained offspring compared with the Ctrl (Fig. 6a–d). These data reveal the molecular mechanism underlying the poor memory improvement observed in 3XTg mice by paternal training. These data also imply that the epigenetic modulation of histone acetylation must be blocked by some unknown factors on 3XTg AD model mice.

## Discussions

Several studies show maternal or paternal stressing experience and the negative impact across generations^5–9, 30^. However, the exclusive effect of father’s experience on the offspring is rarely known, except a recent report shown that stressing fathers underwent in early life was favorable for the offspring^12^. Furthermore, the positive epigenetic transmission is more meaningful to put into practice and brings benefits. To this end, a report showed that spatial training availed on individuals themselves^33^. Our current study is the first to show that the beneficial effects of father’s spatial training can be endowed to their children.

By biochemical analysis, we found that among various memory- or synapse-associated proteins, including CREB, GluR2A, GluR1, GluR2, SYN1 and SYT1, only SYT1 was significantly increased in the F1 offspring after training on fathers. These data suggest the involvement of SYT1 in the facilitated spatial memory of the trained. SYT1 is a presynaptic vesicle integral membrane protein that regulates neurotransmitter release by activating fast synchronous fusion and suppressing slower asynchronous release^20, 34^. It is currently not understood why the training for one week only increases SYT1 but not significantly influence other proteins in the offspring. We speculate the increased SYT1 may initiate a cascade of transmitter release and subsequently synaptic activity, which can be deduced from its enhanced and widespread expression measured by immunohistochemical/immunofluorescence staining. In SYT1 knock-out neurons, number of the docked vesicles, total evoked release, and the release percentage are significantly decreased^35^. Consistent with our findings, physical exercise including moderate treadmill running and voluntary wheel running augment SYT1 level in the hippocampus, while only treadmill exercise augments its expression levels in amygdale. Moreover, SYT1 knockdown in amygdale abolishes the treadmill exercise-facilitated one-trial passive avoidance performance^4^. These data suggest that SYT1 is crucial for synaptic transmission and the formation of learning and memory.

We observed that training increased association of SYT1 promoter and acetylated H3K14 in F1 hippocampus, which suggests that histone acetylation can upregulate SYT1 and thus contribute to the enhanced learning and memory. The increased association of SYT1 promoter and acetylated H3K14 was also seen in father’s sperms, indicating that the augmented histone acetylation in the offspring may be inherited from the trained fathers. In mammals, most histones are replaced by nucleoprotamine during spermatogenesis, but a small fraction of nucleosomes and histones are still retained for modulating embryo development^36^. In mice, the retained nucleosomes are largely composed of histone H3.3 variant and enriched in GC-rich DNA promoter regions^37^. In the present study, we could detect association of acetylated H3K14 and SYT1 promoter only when the primers are designed towards GC-enriched promoter regions. Besides, the target promoter regions are predicted to bind with activated transcription factor 2 (ATF2), which not only interacts with P300/CREB binding protein, but also intrinsicly acetylate histones^38^. This may medidate upregulated acetylation on SYT1 gene.

There is currently no cure for genetic AD, therefore, we tested whether training fathers could attenuate the offspring’s behavioral impairments in 3XTg AD mice. Unfortunately, we did not see significant improvement in this one-week training model when tested the offspring at 7-m old, despite that we observed significant improved memory in 3-m trained fathers. The underlying cause may be the interference of amyloid precursor protein and/or tau protein in the 7-m 3XTg offspring, as 3-m 3XTg AD fathers had the similar cognitive capacity with 3-m wild type fathers. Histone acetylation is a dynamic process regulated by acetyltransferase and deacetylase. Histone deacetylase (HDAC) activity is elevated in the APP/PS1 mouse model, and memory impairments in these animals can be ameliorated by HDAC inhibitors^39^. Moreover, HDAC mediates the decrease in drebrin cluster density on spine induced by amyloid beta oligomers^40^. Therefore, the results observed in Tg mice suggest that the learning ability in F1 may not be paternally inherited, or/and the brain impairment overcomes paternal learning inheritance at an old age, which deserve further investigations.

DNA methylation^41, 42^ and micro RNA^43, 44^ also play important roles in epigenetic transmission in stress models, which may also be involved in our paradigms. Further, the mechanisms underlying the increased histone acetylation in trained 129s F1 offspring may involve acetyltransferase and deacetylase, and we may explore the profound mechanism in future studies.

## Conclusions

In the present study, we found that paternal spatial training for one week facilitates F1 offspring’s spatial memory and synaptic plasticity with augmented histone acetylation on SYT1 promoter, whereas the same training paradigm did not induce significant memory improvement on the triple transgenic AD mouse model.

## Methods

### Animals

3XTg mice were purchased from Jackson Laboratory and bred on a 129/C57BL/6 background in the Experimental Animal Central of Tongji Medical College, Huazhong University of Science and Technology. As a classic AD model^19^, the mice are characteristic of knock-in of the Swedish double mutation of amyloid precursor protein (APPKM670/671NL), a presenelin 1 mutation (PS1M146V), and a frontotemporal dementia tau mutation (tauP301L). After genotyping, 3XTg and wild-type mice were raised at same conditions under a 12:12 h light-dark cycle with free access to food and water. All animal experiments were performed according to the “Policies on the Use of Animals and Humans in Neuroscience Research” revised and the animal study was allowed by the Academic Review Board of Tongji Medical College.

### Spatial training paradigm and procedures

The spatial training paradigm was conducted using Morris water maze (MWM) procedure^45^. Mice were trained to search a hidden platform (~2 cm under water) in the pool for 6 days. Mice learned 3 trials (with a 30 min interval) from different entrance of 3 quadrants except for target quadrant from 2:00 PM to 6:00 PM. Mice were placed towards the inner wall of pool, which is attached with different signs, then into the water by hand. No more than 60 s was permitted for each mouse to seek the platform, after which 30 s’ residence on the platform was necessary for learning. The swimming path and time to locate the platform (latency) were recorded by a camera suspended 1.5 m above the water using Noldus video tracking system (Ethovision).

The trained 3XTg and 129 wild-type male mice (3-m old) were mated respectively with untreated 3XTg and 129 wild-type females. As control, the 3XTg or 129 wild-type mice were swum for 6 days in water without training, and then they were mated with untreated females. 6~10 males were trained or swum in each group and those behaved well were selected to breed. After birth, F1 offspring were bred under the same maintenance till grew up. The spatial capacity of offspring was tested by MWM when they were 7-m old. After 6 days learning trials, the spatial memory was detected at day 8 without platform. In the adjacent days, Electrophysiological analysis (LTP), Western blotting (WB), Immunohistochemistry (IHC), mRNA extraction, and Chromatin immunoprecipitation (ChIP) were performed on offspring from four groups. To illuminate experiment procedures, we drew flow diagrams in Figs 1a and 4a.

### Fear conditioning

After the MWM training or swimming, 7~10 fathers from four groups were assigned to contextual fear conditioning to test effect of spatial training on hippocampus-dependent associative memory. On the first day, mice were placed in the conditioning chamber (width × depth × height; 175 × 165 × 300 mm; with a floor of 26 steel rods) for 3 min, then were subjected to 3 times unsignalled foot-shocks (once a minute, 0.8 mA, 2-s duration, and 1 min apart). After the last shock, mice were returned to their home cages. 24 hours later, mice were placed back into the conditioning chamber for 3 min without foot-shocks and the total freezing time was recorded. Each time before mice were placed into the chamber, the chamber was cleaned with 70% ethanol. After fear conditioning, mice were no longer used for other experiments.

### Electrophysiological analysis

Animals were anesthetized and cut into horizontal sections every 350 μm in ice-cold artificial cerebrospinal fluid (aCSF). The aCSF is composed as following: 126 mM NaCl, 3 mM KCl, 1.25 mM NaH_2_PO_4_, 24 mM NaHCO_3_, 2 mM MgSO_4_, 2 mM CaCl_2_, and 10 mM glucose. Sections were transferred rapidly and gently to a beaker filled with aCSF where was piped in oxygen continuously. After incubated 90 min, sections were transferred to a 8 × 8 array of planar microelectrode, each 50 × 50 μm in size, with an interpolar distance of 150 μm (MED-P515A; Alpha MED Sciences, Kadoma, Japan) and dipped in flowing aCSF and oxygen with covering of nylon net pasted onto a platinum loop. We located sections to make sure that CA1 and CA3 were exactly within the 8 × 8 array. The Input/Output curves of fEPSP amplitudes were recorded at CA1 pyramidal neurons along with increasing stimulation intensity in CA3 pyramidal neurons. When fEPSP amplitudes of I/O curve reached a plateau, the stimulation intensity which evoked 40% of the plateau amplitude in each slices was took as test pulse. Long term potentiation (LTP) was acquired in CA1 through 3 traces of high frequency stimulation (HFS; 100 Hz, 1 s duration) of the Schaeffer collateral-commissural fibers in CA3 using the MED64 System (Alpha MED Sciences).

### Western blotting

Western blotting was performed according to methods established in our laboratory^46^. Hippocampus was removed and homogenized on ice with RIPA buffer (Bi Yuntian). The samples were separated by SDS-PAGE, transferred onto nitrocellulose membranes and then covered by 5% non-fat milk. After washings by Tween-TBS, the membranes were detected by primary antibodies overnight, and then by secondary antibodies conjugated to IRDyeTM (800CW) for 1 h. The visual blots were acquired from the Odyssey Infrared Imaging System (Licor biosciences, Lincoln, NE, USA). The full length blots are presented in Supplementary Information. The primary antibodies are displayed as follows: CREB (cell signaling, 1:1000), p-CREB (cell signaling, 1:1000), BDNF (SANTA CRUZ, 1:1000), GluN2A (abcam, 1:1000), GluN2B (abcam, 1:1000), GluR1 (Millipore, 1:500), GluR2 (Millipore, 1:500), PSD95 (cell signaling, 1:1000), anti-Synaptotagmin1 (abcam, 1:2000), anti-Synapsin1 (Millipore, 1:1000), PT231 (SAB, 1:500), PP1 (Millipore, 1:200), HT7 (Thermo, 1:500), β-actin (abcam, 1:1000), acetylated H3 (Millipore, 1:1000), acetylated H3K14 (Millipore, 1:1000), acetylated H3K9 (Millipore, 1:1000), Histone 3 (abcam, 1:1000).

### Immunohistochemistry and Immunofluorescence

Brains were removed after anesthetized and perfused by 4% paraformaldehyde. The brains were post-fixed in perfusate for 24 h and dehydrated in 30% sucrose-PBS for 48 h. After embedding, brains were cut into 25 μm coronal sections. For immunohistochemistry (IHC) and immunofluorescence (IF), the experiments were carried out according to the established methods^47^. The floating sections were incubated with the same primary antibodies (anti-Synaptotagmin1, abcam, 1:200). For IHC, the sections were incubated the same time with diaminobenzidine tetrachloride system. The images were observed using a microscope (Nikon, Tokyo, Japan). For IF, the images were captured using a Carl Zeiss LSM710 confocal microscope.

### Quantitative RT–PCR

Hippocampus was homogenized in reagent Trizol (Invitrogen) using a Teflon glass homogenizer. Reverse-transcription was conducted using TransScript II First-Strand cDNA Synthesis (Takara). Afterwards, qPCR was conducted with BioRadC1000 machine (BioRad Laboratories, Inc, USA). The system included 0.5 μM forward and reverse primers, 20 μl SYBR Green PCR master mix (containing MgCl_2_) and 1 μl cDNA. The primers (Invitrogen) were projected as follows: SYT1: sense 5′ CCATAGCCATAGTTGC 3′, anti-sense 5′ GTTTCAGCATCGTCAT 3′, β-actin: sense 5′ GAGACCTTCAACACCCCAGC 3′, anti-sense 5′ GGAGAGCATAGCCCTCGTAGAT 3′.

### Agarose gel electrophoresis

The q-PCR products were collected for agarose gel electrophoresis. The agarose gel is dissolved in TBE buffer (890 mM Tris-borate and 20 mM EDTA) and the final concentration is 1%.

### Chromatin immunoprecipitation assay

Chromatin immunoprecipitation (ChIP) was carried out as described (#17-371, upstate, cell signaling, USA). Hippocampus was cut into pieces on ice. The pieces were incubated with 37% formaldehyde for 10 min to enhance crosslinking, followed by 10 × Glycine to end the process. After 5 times washed with PBS and centrifuged 5 min at 700 × g at 4 °C, the sediment was homogenized with SDS (contains 200 × cocktail) using the glass homogenizer and the supernatants were obtained through centrifuging 5 min at 12,000 g at 4 °C. The supernatants were sonicated on ice using a Digital Sonifier with 5 cycles of 5 s sonication and 10 s intervals at 30% power setting. Each sample was immunoprecipitated rotationally overnight at 4 °C with 10 μg of the primary antibodies (H3K14ac). As input, nothing was added into one sample while anti-RNA polymerase II into another sample as positive control and mouse IgG into the third sample as negative control. Afterwards, the systems were incubated rotationally with 60 μl Protein G Agarose for 1 h at 4 °C. Through brief centrifugation (5000 g for 1 min), the Protein G Agarose-histone-DNA complexes were enriched in the sediment. Soon, the complexes were washed three times in sequence with low salt buffer, high salt buffer, LiCl buffer and extra washed twice with TE buffer. The complexes were then eluted by Elution Buffer (20% SDS, 1M NaHCO_3_) and supernatants (histone-DNA complex) were collected by brief centrifugation. The complexes were disassociated with 5M NaCl overnight at 65 °C. Then, the DNA was purified with RNase A for 30 min at 37 °C and Protein K for 1 h at 45 °C. To quantify histone-associated DNA, qPCR was performed with following primers: SYT1 (targeting upstream from the transcription start site in the promoter: +433~+492): sense 5′ ACCTCTGCGCTTCAATCT 3′, anti-sense 5′ TTCTCACAATAGGCTTACGG3′, β-actin: sense 5′ GAGACCTTCAACACCCCAGC 3′, anti-sense 5′ GGAGAGCATAGCCCTCGTAGAT 3′.

### Statistical analyses

The data were analyzed by unpaired t-test for two-group comparisons except that escape latency in the MWM and I/O curve of the LTP were analyzed by two-way ANOVA followed by post hoc tests (Prism 6; GraphPad Software). The data were presented as mean ± SEM and P < 0.05 was accepted as statistically significant.

## Electronic supplementary material

Supplementary Information
