# Supplementary material for: Paternal spatial training enhances offspring’s cognitive performance and synaptic plasticity in wild-type but not improve memory deficit in Alzheimer’s mice
Source: Sci Rep. 2017 May 8;7:1521. doi: 10.1038/s41598-017-01811-3 (PMC5431522; doi:10.1038/s41598-017-01811-3)

Paternal spatial training enhances offspring's cognitive performance and synaptic plasticity in wild-type but not improve memory deficit in Alzheimer's mice

Shujuan Zhang[a,1], Xiaoguang Li[a,1], Zhouyi Wang[b,1], Yanchao Liu[a], Yuan Gao[a], Lu Tan[c], Enjie Liu[a], Qiuzhi Zhou[a], Cheng Xu[a], Xin Wang[a], Gongping Liu[a], Haote Chen[b,]*, Jian-Zhi Wang[a,d,]*.

SUPPLEMENTARY INFORMATION

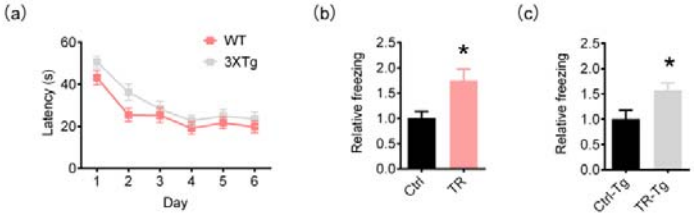

Supplementary Fig. 1 | Spatial training improves individual hippocampus-dependent memory in wild type (WT) and triple transgenic (3XTg) paternal mice.

(a) The MWM learning curve of 3-m WT and 3XTg fathers: escape latency to find the hidden platform in the maze during 6 days training. (two-way ANOVA row factor, $F_{5, 424}$=19.32, p<0.0001; Bonferroni post hoc tests, n.s, n=9~10 per group).

(b) Contextual fear conditioning of swum (Ctrl) and trained (TR) WT fathers: quantitative analyses of freezing response measured at 24 h after fear training. (*p<0.05, two-tail t-test, n=7~9 per group).

(c) Contextual fear conditioning of swum (Ctrl-Tg) and trained (TR-Tg) 3XTg fathers: quantitative analyses of freezing response measured at 24 h after fear training. (*p<0.05, two-tail t-test, n=9~10 per group).

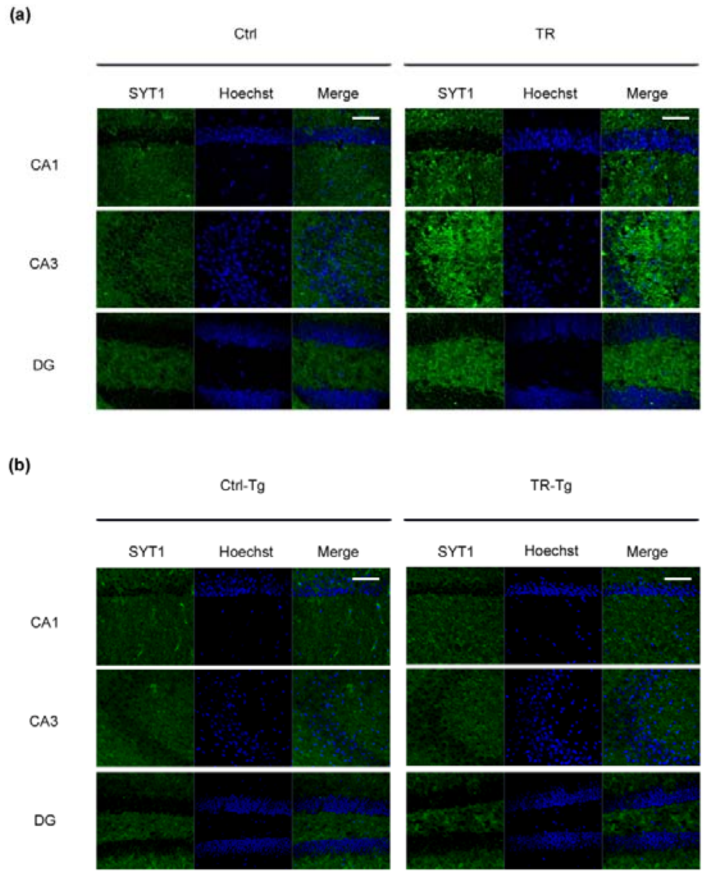

Supplementary Fig. 2 | Father's spatial training augments synaptotagmin 1 in 129s F1 offspring but not in 3XTg F1 offspring.

(a) Immunostaining of SYT1 in hippocampus of 7-m 129s F1 offspring brain slices.

The scale bar=5 μm.

(b) Immunostaining of SYT1 in hippocampus of 7-m 3XTg F1 offspring brain slices.

The scale bar=5 μm.

Supplementary primary blots

Fig.2a

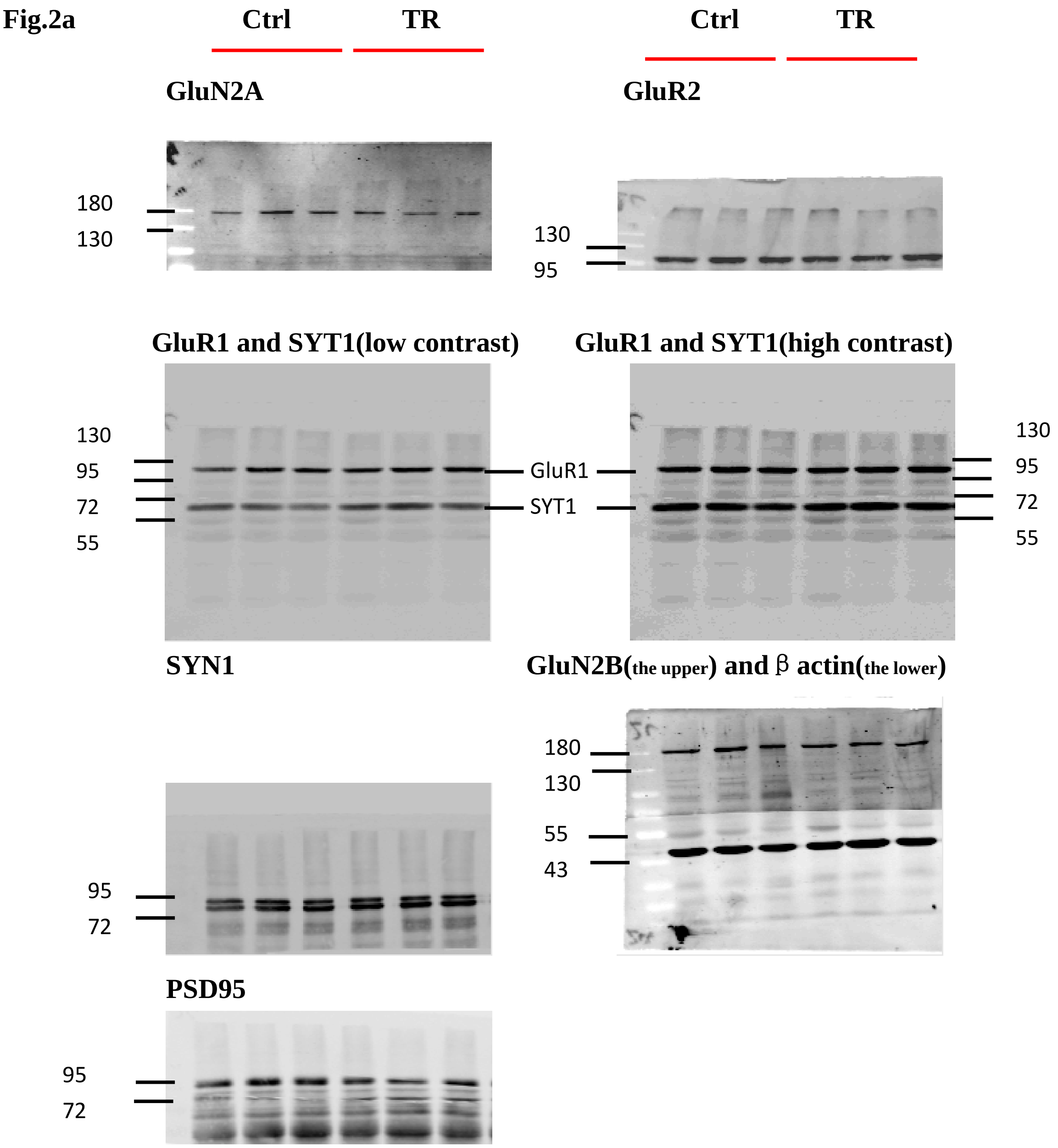

Fig.2e

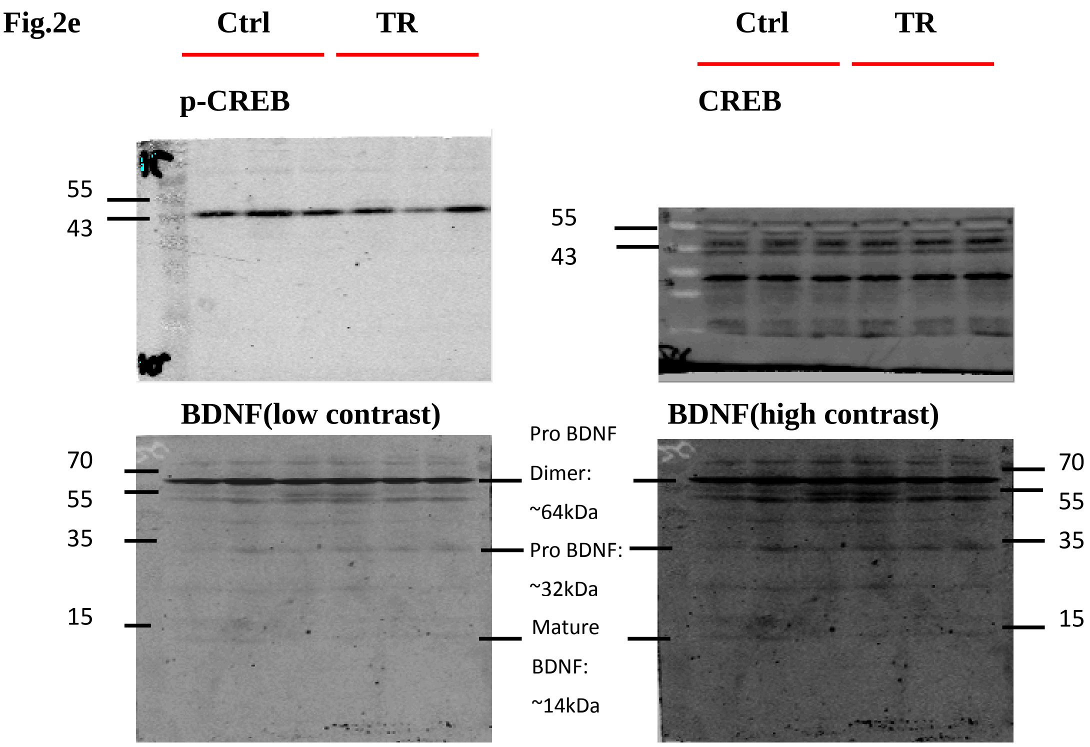

Fig.2f

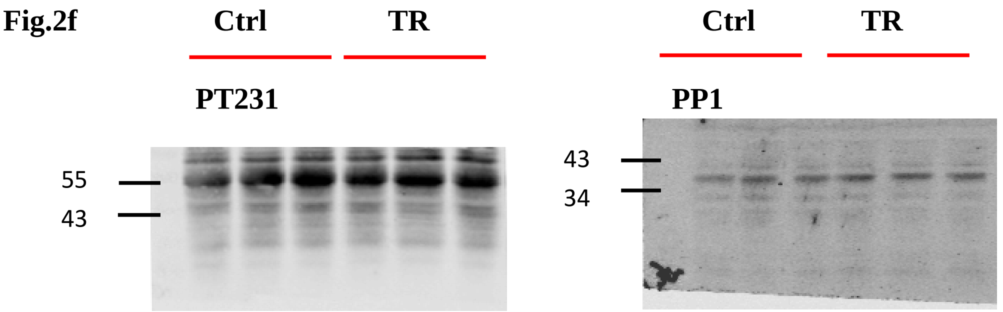

Fig.3a

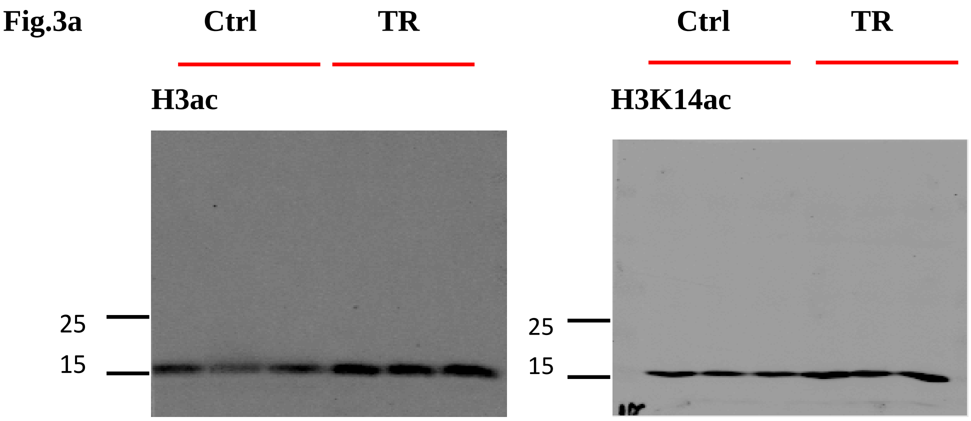

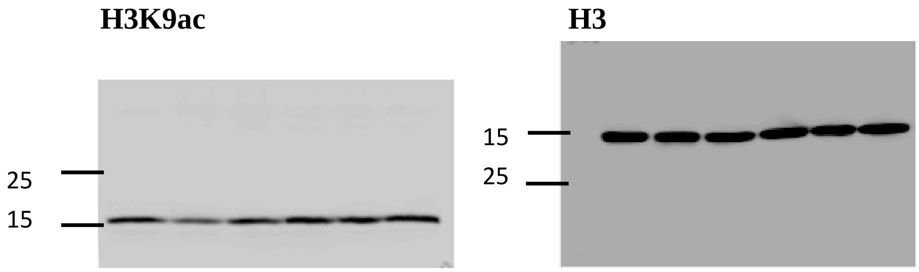

H3K9ac
H3
25
15
15
25

Fig.5a

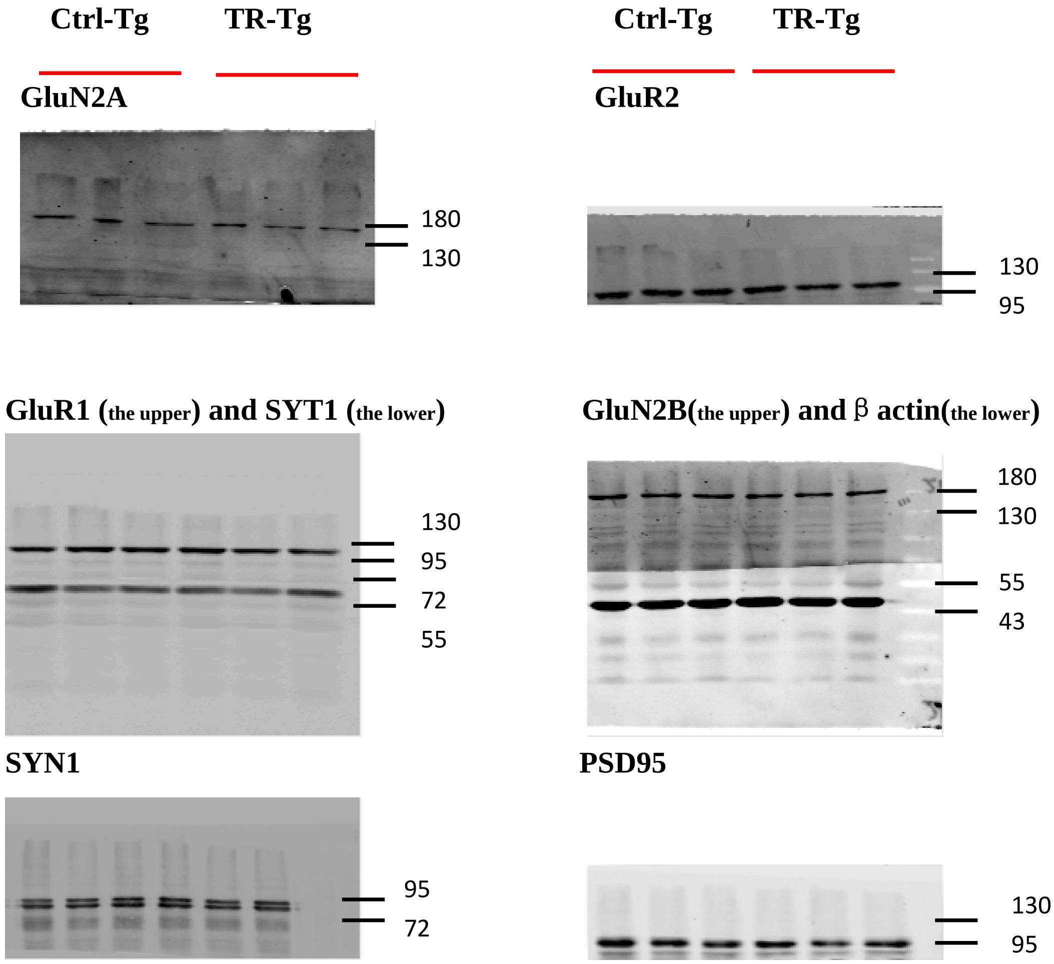

Ctrl-Tg
TR-Tg
Ctrl-Tg
TR-Tg
GluN2A
180
130
GluR2
130
95
GluR1 (the upper) and SYT1 (the lower)
130
95
72
55
GluN2B(the upper) and β actin(the lower)
180
130
55
43
SYN1
95
72
PSD95
130
95

Fig.5c

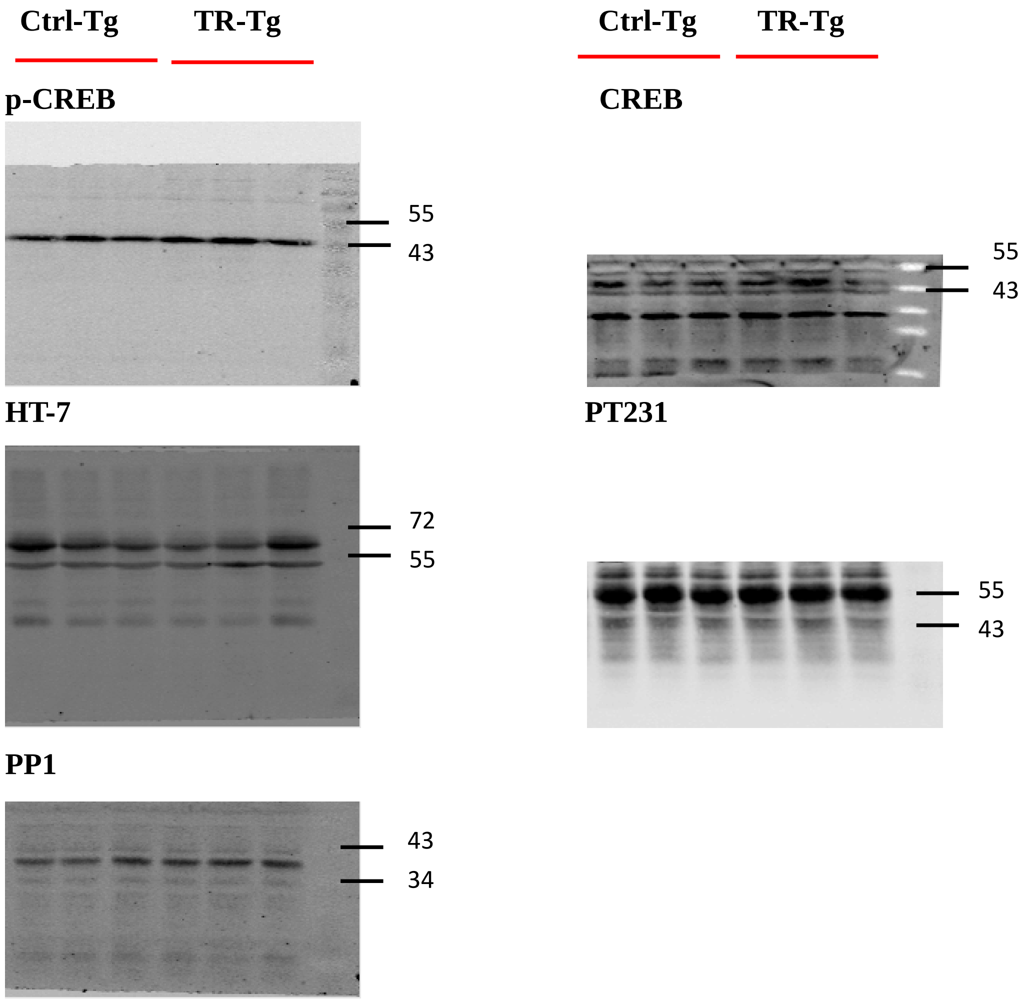

Ctrl-Tg
TR-Tg
p-CREB
55
43
Ctrl-Tg
TR-Tg
CREB
55
43
HT-7
72
55
PT231
55
43
PP1
43
34

Fig.6a

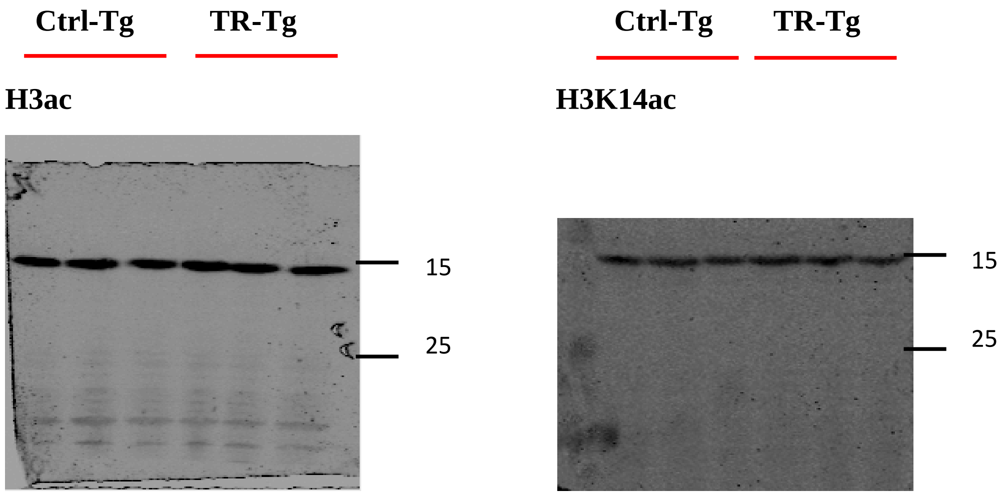

Ctrl-Tg
TR-Tg
H3ac
15
25
Ctrl-Tg
TR-Tg
H3K14ac
15
25

H3K9ac

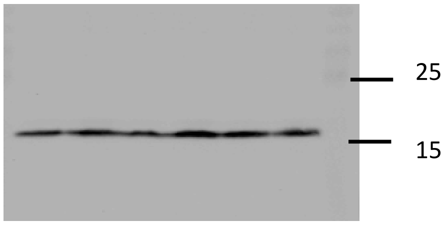

H3

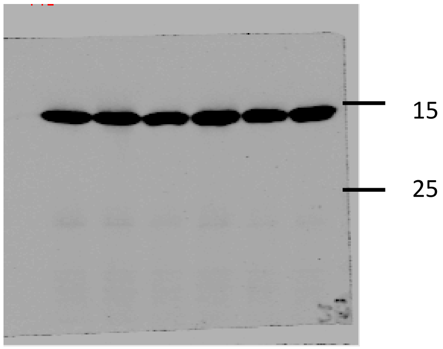

Supplement: Supplementary file 1 — Supplementary Information [file 41598_2017_1811_MOESM1_ESM.pdf]
